# A Global, High-Resolution Index of Risk and Exposure of Humans to Ticks, Lyme Borreliosis, and Crimean–Congo Haemorrhagic Fever

**DOI:** 10.3390/pathogens15090944

**Published:** 2026-09-05

**Authors:** Agustín Estrada-Peña

**Affiliations:** Department of Animal Health, Faculty of Veterinary Medicine, University of Zaragoza, 50013 Zaragoza, Spain; antricola@me.com

**Keywords:** ticks, *Borrelia*, Crimean–Congo haemorrhagic fever, global indicators, hazard, exposure, vulnerability

## Abstract

Ticks are among the most important arthropod vectors affecting human and animal health worldwide. We present a set of globally harmonized indicators integrating hazard, exposure, and vulnerability associated with human-biting ticks and with two major tick-borne pathogens, *Borrelia burgdorferi* sensu lato (Lyme borreliosis), and *Orthonairovirus haemorrhagiae* (Crimean–Congo haemorrhagic fever virus, CCHFV). Hazard indices were derived from the distributions of confirmed vector tick species. Exposure was incorporated through rural human population density, livestock density, and the abundance of competent vertebrate reservoirs and/or amplification hosts, as applicable. Vulnerability was assessed using country-level indicators related to healthcare accessibility, socioeconomic development, and demographic dependency. The indices show spatially structured global patterns. Tick hazard is concentrated in eastern North America, Europe, Central and East Asia; Africa displays fragmented but substantial hotspots. The *B. burgdorferi* s.l. index is restricted to the Northern Hemisphere, following the distribution of *Ixodes* spp. and associated reservoir communities, with highest exposure in Europe and North America. The CCHFV index is confined to the Old World, extending from the Mediterranean region through the Middle East and Central Asia into Sub-Saharan Africa, reflecting the distribution of *Hyalomma* ticks and ungulates. The vulnerability indicators highlight regional patterns of high exposure and limited adaptive capacity, particularly in Sub-Saharan Africa and parts of the Middle East. Our framework provides the first global set of harmonized, high-resolution indices integrating vector ecology, reservoir communities, exposure, and vulnerability for major tick-borne diseases of humans. It offers a flexible platform for comparative epidemiology, surveillance prioritization, and public health decision-making at multiple administrative scales.

## 1. Introduction

Ticks are obligate hematophagous ectoparasites of major medical and veterinary importance, acting as vectors of a wide diversity of pathogens with complex life cycles. The epidemiology of tick-borne diseases is inherently multi-scalar and emerges from the interaction between environmental suitability, host community composition, vector population dynamics, and pathogen circulation [1]. Within this framework, a comprehensive understanding of tick-borne disease systems requires the integration of ecological processes with human behavioural and socio-environmental drivers.

From an eco-epidemiological perspective, transmission risk stems from the spatiotemporal co-occurrence of vectors, competent vertebrate hosts, and pathogen reservoirs in environmentally suitable landscapes. Climatic conditions define the fundamental niche of ticks and strongly constrain their geographic distribution, seasonal activity, and survival, while indirectly shaping host availability and community structure. At the same time, climate and land-use patterns influence human exposure by modulating the extent of overlap between human activities and tick habitats. Consequently, changes in climatic suitability can alter not only vector abundance but also the synchrony and connectivity among hosts and pathogens, thereby reshaping transmission dynamics across scales [2,3,4].

Within this context, effective risk assessment remains challenging due to fragmented surveillance systems and the lack of harmonized definitions distinguishing hazard from risk across disciplines, limiting comparability and decision-making [5,6]. Moreover, the impact of tick-borne diseases is ultimately mediated by the vulnerability of exposed populations, which encompasses the capacity of individuals and health systems to anticipate, cope with, and recover from adverse outcomes, as conceptualized in broader climate–health frameworks [7,8].

Current health risks from ticks and tick-borne pathogens stem from the continued presence of ticks in endemic areas, the presence of key vertebrate hosts that harbour large numbers of ticks and the co-occurrence of pathogen reservoirs that facilitate transmission to humans [9]. A suitable climate niche supports these permanent populations of both ticks and their vertebrate hosts. This climate can also influence the social habits linking human and tick habitats [2,10]. Therefore, tick-related health issues result from multiple factors that promote the persistence and abundance of ticks and human contact with them [11].

Monitoring and evaluation are crucial for managing infectious disease risks in regions lacking harmonised definitions of hazard and risk [12,13]. Consequently, indicators of hazard by vectors must consider who is exposed, the conditions of exposure (e.g., co-existence of reservoirs that contribute to enhancing the circulation of a pathogen) and vulnerability. Vulnerability is typically defined as the capacity of exposed individuals and healthcare institutions to avoid, prepare for, cope with and recover from impacts [14,15]. Currently, no harmonised hazard index has yet been established [16].

Despite substantial advances in modelling vector distributions and disease risk, the operationalization of these concepts into actionable indicators remains limited. In particular, the lack of harmonized definitions distinguishing hazard, exposure, and risk across disciplines, together with fragmented surveillance systems, constrains the comparability and applicability of existing approaches [5,6,12,13,17]. Moving beyond vector presence alone requires incorporating the ecological context of pathogen circulation, including reservoir communities and host–vector interactions.

Furthermore, the public health impact of tick-borne diseases is mediated by the vulnerability of exposed populations, understood as the capacity of individuals and health systems to anticipate, cope with, and recover from adverse outcomes. This concept aligns with broader climate–health frameworks, where vulnerability emerges from the interaction between exposure, sensitivity, and adaptive capacity [7,8,14,15]. However, globally consistent indicators that integrate these components into a coherent framework for tick-borne diseases remain largely undeveloped.

This study builds on existing data about human-biting ticks to create an index of tick hazard in humans. It also develops corresponding indexes for two major tick-borne pathogens: *Borrelia burgdorferi* s.l. (Bb), the etiological agent of Lyme disease, and *Orthonairovirus haemorrhagiae*, formerly known as Crimean–Congo haemorrhagic fever virus (CCHFV). These indexes cover the entire world at the smallest administrative division level (GADM) for greater resolution and reliability, ultimately forming country-specific indices. We selected *Borrelia burgdorferi* s.l. and CCHFV as model pathogens because they represent two contrasting, high-consequence transmission systems with distinct eco-epidemiological paradigms: a Northern Hemisphere system driven by *Ixodes* vectors and vertebrate reservoirs *versus* an Old World system driven by *Hyalomma* vectors and ungulate amplification hosts. Applying a single, harmonized macro-ecological framework to these fundamentally different systems allows us to demonstrate the versatility and robustness of multi-trophic hazard and exposure indexing across disparate global health contexts. The complete data is publicly available for further improvements and refinements.

## 2. Materials and Methods

The workflow of this study follows three well-defined steps: (a) the derivation of the tick-borne hazard, obtained by modelling the distribution of human-affecting ticks or vectors transmitting *Borrelia burgdorferi* s.l. (Bb) or Crimean–Congo haemorrhagic fever virus (CCHFV); (b) the derivation of exposure, based on the abundance of key vertebrate reservoirs, amplifying hosts, and human demographic factors; and (c) the evaluation of vulnerability.

Hazard was defined following the United States Environmental Protection Agency guidelines (https://www.epa.gov/risk (accessed on 7 March 2025)) as a factor or exposure with potential adverse health effects, or the probability of occurrence of a harmful event. Exposure is conceptualized here as the contact of human populations with ticks (either as parasites *per se* or as vectors of pathogens) or the presence and abundance of key competent reservoirs. The vulnerability to ticks or tick-borne pathogens within a territory is the summation of all risk and protective factors that collectively determine whether a region experiences adverse health outcomes [18,19].

All the aforementioned indices vary across spatial and temporal scales in response to changes in economic development, social capital, population demographic structures (such as the proportion of elderly people or the degree of urbanization), trade and travel patterns, the prevalence of pre-existing medical conditions, and acquired factors such as immunity, as well as genetic and other determinants [20].

### 2.1. Calculation of the Tick Hazard Index

We considered a set of species of ticks that are reported as frequent parasites of humans [21]. The species included in the derivation of the Tick Hazard Index (THI) were *Ixodes ricinus*, *Ixodes persulcatus*, *Ixodes nipponensis*, *Ixodes scapularis*, *Ixodes pacificus*, *Ixodes cookei*, *Ixodes uriae*, *Ixodes holocyclus*, *Ixodes pavlovskyi*, *Ixodes ovatus*, *Amblyomma americanum*, *Amblyomma cajennense* s.l., *Amblyomma triguttatum*, *Amblyomma tigrinum*, *Amblyomma variegatum*, *Amblyomma hebraeum*, *Dermacentor andersoni*, *Dermacentor occidentalis*, *Dermacentor variabilis*, *Dermacentor albipictus*, *Dermacentor marginatus*, *Dermacentor reticulatus*, *Dermacentor nuttalli*, *Dermacentor silvarum*, *Hyalomma aegyptium*, *Hyalomma anatolicum*, *Hyalomma asiaticum*, *Hyalomma dromedarii*, *Hyalomma albiparmatum*, *Hyalomma glabrum*, *Hyalomma impressum*, *Hyalomma marginatum*, *Hyalomma rufipes*, *Hyalomma excavatum*, *Hyalomma scupense*, *Hyalomma turanicum*, *Hyalomma truncatum*, *Rhipicephalus bursa*, *Rhipicephalus sanguineus* s.l., and *Rhipicephalus turanicus*.

Field records for these species were obtained from open-access compiled datasets [22,23]. All the details underpinning the compilation of the dataset are available in the mentioned reference. Data used for this compilation were obtained from FigShare at the link https://doi.org/10.6084/m9.figshare.32532780 (accessed on 1 December 2025).

For this study we managed to assemble a set of 87,914 records of ticks biting humans worldwide that were used to develop individually modelled maps for each species, producing global environmental suitability surfaces (see below, point 2.1.2). Suitability values were subsequently re-scaled to a 0–100 range using a linear transformation, representing the hazard values. The calculated raster values of tick suitability were transferred to administrative regions using standard GIS spatial routines in QGIS (http://www.qgis.org, accessed on 1 December 2025). The geometric mean (excluding zero values) of the probabilistic pixels representing each species within each GADM division was calculated to derive the final THI for that specific administrative unit, formulated as follows:

$$THI_{i}={\left(\prod_{s\in S_{i}}H_{is}\right)}^{\frac{1}{\left|S_{i}\right|}}$$

where *S_i_* represents the subset of tick species present in the GADM administrative unit *i* with suitability values greater than zero (*H_is_* > 0), ∣*S_i_*∣ denotes the total number of species included in that specific subset, and *H_is_* is the rescaled suitability value of species *s* within the region *i*.

#### 2.1.1. Explanatory Variables

Environmental predictors consisted of a set of climate-derived variables representing temperature and atmospheric moisture conditions. These predictors were extracted at occurrence locations from raster layers to build the training datasets. Climatic data were obtained from the TerraClimate repository at a 4 km spatial resolution. Monthly data for maximum temperature (TMAX), minimum temperature (TMIN), and vapor pressure deficit (VPD) were compiled across the study area for the period 1995–2020 at monthly intervals. VPD quantifies the deficit of water vapor in the air and is tightly linked to relative humidity and tick desiccation stress. Harmonic regression analysis [23] was performed on the monthly averages across the complete time series, independently for each climate variable, to capture pure seasonal profiles.

The harmonic regression is defined as follows:

$$f(t)=a_{0}\;+\;\underset{n=1}{\overset{N}{\sum }}\left[a_{n}cos\left(\frac{2\pi nt}{T}\right)\;+\;b_{n}sin\left(\frac{2\pi nt}{T}\right)\right]$$

where *_a_*_0_ is the offset term representing the average of the signal; *N* is the maximum number of harmonics used for the regression; *a_n_* and *b_n_* are the Fourier coefficients determining the amplitude of each wave; *t* is the time variable in months; and *T* is the fundamental period of the data, corresponding to a complete 12-month annual cycle.

Harmonic regression was executed for TMAX, TMIN, and VPD. The retention of the first five coefficients per variable yielded a total of 15 climatic predictors. All climatic calculations were executed in R (v.4.0.2, R Core Team, Vienna, Austria) using the terra package (v.1.8-60, [24]).

#### 2.1.2. Modelling: Random Forest

Species distributions were modeled independently using the Random Forest (RF) algorithm, an ensemble machine learning method widely utilized in ecological niche modelling due to its high predictive performance and capacity to capture complex, non-linear relationships without parametric assumptions [25]. This makes it highly robust when true absence data are unavailable and sampling effort is heterogeneous.

Random Forest models were implemented by generating multiple classification trees from bootstrap samples of the training data, subsampling a random subset of predictors at each node split. This procedure minimizes overfitting and enhances generalization. For each species, the continuous model output represents a relative habitat suitability score (a relative classification probability across space) rather than a strictly calibrated probability of occurrence.

To mitigate the inflating effects of spatial autocorrelation and geographical sorting bias, model performance was evaluated using a spatial block validation framework. Occurrence records were partitioned into geographically independent training and testing subsets using spatially structured blocks. In all iterations, approximately 70% of the spatial blocks were used for model training, while the remaining 30% were held out for validation.

To account for potential target-group sampling biases, pseudo-absences were randomly allocated outside a minimum spatial buffer from presence points, maintaining a prevalence ratio of 1:1, which optimizes Random Forest classification performance under spatial block validation [26]. Model performance was evaluated using multiple complementary validation metrics calculated from the holdout spatial blocks: sensitivity, specificity, omission rate, and the threshold that maximizes the sum of sensitivity and specificity (True Skill Statistic threshold split). Boyce Index was employed to evaluate continuous suitability curves without binarization constraints, measuring whether observed presences occur more frequently in areas predicted as highly suitable compared to a random distribution.

The classification threshold is a scalar t ∈ [0, 1] used to convert continuous model outputs into binary predictions. The omission rate represents the proportion of observed presences incorrectly predicted as absences (false negatives). Specificity represents the proportion of reference negatives (absences) correctly classified, with commission error quantified as 1—specificity, whereas sensitivity represents the proportion of correctly predicted presences. Accuracy describes the total proportion of correctly classified instances. Models were separately developed for each species with different combinations of the explanatory variables; the examination of the performance indices for each species-model pair guided the selection of the optimal combination of explanatory variables. See validation of the models (point 2.6) for the protocols of validation.

### 2.2. Calculation of Hazard by Borrelia burgdorferi s.l. (The Bb Hazard Index)

We are aware of maps displaying the risk of *Borrelia burgdorferi* s.l. (Bb) in parts of North America and Europe (e.g., [27]). These maps are commonly based on the gold standard of pathogen prevalence in questing nymphal ticks, or the density of infected nymphs (DIN). Establishing such standardized values can be particularly problematic for territories where data are primarily available at the national level, which is insufficient to fulfill the spatial resolution required for smaller administrative divisions. Furthermore, a proportion of published studies may lack basic quality control regarding reported associations between pathogens and ticks, resulting in spurious data about pathogen prevalence in vectors.

We approached the derivation of the Bb Hazard Index using the modeled spatial suitability of ticks with confirmed vector competence for transmitting the pathogen to humans or to circulate Bb among the reservoirs. The species included were *I. ricinus*, *I. persulcatus*, *I. pavlovskyi*, *I. ovatus*, *I. nipponensis*, *I. scapularis*, *I. pacificus*, *I. cookei*, and *I. uriae*. We calculated the Bb Hazard Index for each GADM unit as the geometric mean of the suitability values of these selected tick vector species. If the value was higher than 0, it was entered in the calculation of the geometric mean; a value = 0 was removed from the calculations because geometric mean does not admit zeroes.

### 2.3. Calculation of Hazard by Crimean–Congo Haemorrhagic Fever Virus

Similar methodological challenges surround the calculation of the CCHFV hazard, primarily driven by a substantial lack of systematic data regarding prevalence in questing ticks. Furthermore, the literature data are often confounded by erroneous vector identifications and by PCR analyses conducted on engorged, feeding ticks. The latter introduces false positives due to the contamination of ticks by the blood of viraemic hosts, incorrectly assigning the category of “new vectors” in published reports.

We based the construction of the CCHFV Hazard Index on the distribution of tick species with confirmed vector competence supported by experimental transmission studies or robust ecological inference (summarized in [28,29]). The included ticks were *A. variegatum*, *A. hebraeum*, *R. bursa*, *H. aegyptium*, *H. turanicum*, *H. truncatum*, *H. scupense*, *H. rufipes*, *H. marginatum*, *H. excavatum*, *H. dromedarii*, *H. asiaticum*, and *H. anatolicum*. All selected species have been documented either biting humans and viraemic mammals or actively participating in virus circulation among infected hosts. The CCHFV Hazard Index was calculated as the geometric mean of suitability values for all the aforementioned species within each GADM unit, with the same comments as above for Lyme disease. The validation of the model for CCHFV was performed separately, including only the species of ticks involved in the transmission of the virus (see point 2.6).

### 2.4. Calculation of Exposure

In epidemiological risk frameworks, hazard indices must be weighted by exposure conditions. The data used to estimate exposure to ticks and their transmitted pathogens included: (a) rural human population density in proximity to tick habitats; (b) livestock density within each GADM unit, serving as a proxy for tick population amplification and direct human-vector contact interfaces; (c) the geographic presence of competent reservoir vertebrates for *B. burgdorferi* s.l. [30]; and (d) the presence of vertebrate hosts capable of amplifying CCHFV infections [30,31].

Estimates of rural human population density (habitants per square kilometer) were sourced from the NASA Socioeconomic Data and Applications Center (SEDAC), accessed via the UNCCD knowledge platform in February 2025.

To refine exposure metrics for Bb, we integrated global occurrence datasets of rodents and birds known to act as competent reservoirs. In regions where vector ticks occur but competent reservoirs are completely absent, exposure to the pathogen is functionally non-existent (i.e., equals zero). For the Palearctic region, we leveraged the complete set of geographic range maps from the Mammal Diversity Database (MDD v1.2) taxonomy, (https://doi.org/10.5281/zenodo.7394529; accessed on 1 December 2025), as well as the “Species habitat suitability of European terrestrial vertebrates for contemporary climate and land use” a set of data available at https://portal.geobon.org/ebv-detail?id=84&v=1, accessed 10 July 2025) [32]. The R package MDD (v1.2) was utilized for the systematic selection of mammalian taxa. Given the absence of a fully harmonized global bird distribution grid, Palearctic avian distributions involved in Bb transmission were extracted from modelled datasets [22]. For the Nearctic region, distribution boundaries were obtained from the USGS Gap Analysis Project (GAP, https://www.usgs.gov/programs/gap-analysis-project, accessed on 1 September 2025), selecting species confirmed as Bb reservoirs within the territory.

We also accounted for the presence of large wild ungulates and livestock. These vertebrates serve as primary hosts for adult tick life stages; they do not amplify Bb but significantly boost tick population dynamics by facilitating female engorgement and subsequent oviposition. On the other hand, large ruminants are deeply involved in the circulation of CCHFV [31] resulting in stable, abundant subsequent tick generations that amplify hazard levels. Wild ungulate distributions were extracted from the aforementioned mammalian databases [32]. For livestock densities (cattle, sheep, goats, and horses per square kilometer), data were obtained from the FAO Global Livestock Environmental Assessment Model (GLEAM) toolbox (https://www.fao.org/gleam/en/, accessed on 2 November 2025). Because livestock densities were strongly right-skewed across regions, data were normalized and rescaled into a 0–100 range.

Human and livestock raster layers were aggregated into GADM administrative units using spatial zonal statistics in QGIS. Wild host distributions, available as binomial expert-validated range polygons, were assigned to GADM units as binary presence/absence (1/0) values. Final indices for Bb and CCHFV exposure involved the multiplication and normalization of these underlying surfaces (human density, vector suitability, livestock abundance, and reservoir presence) to yield uniform, comparable global exposure metrics.

Therefore, the calculated exposure to Lyme disease is

$$LDE={LD}_{th}\times HumanRP\times DenLives\times PresWild$$

where:

LDE: Lyme disease exposure.

LDth: Lyme disease tick hazard (the average of suitability for the tick vectors of *Borrelia* spp.).

HumanRP: Density of human rural population.

DenLives: Density of livestock population.

PresWild: Average of the presence/absence (binary) of reservoirs of the bacterium, resulting in a decimal number in the range 0–1.

Normalized to 0–100 by a sigmoid function with the sigmoid parameters as described below. The sigmoid function (or logistic curve) is an ideal mathematical tool for this task, as it transforms any range of continuous values into a smooth “S-shaped” curve strictly bounded between 0 and 100.

$$f\left(x\right)=100/\left(1+e^{\left(-k\left(x-x_{0}\right)\right)}\right)$$

where the two main parameters are:

x0: (Inflection Point/Center): The original data value at which the function returns exactly 50% (50).

k (Steepness/Rate of Change): Controls how steep the S-curve is. A very small k value results in a smooth, gradual transition between 0 and 100, whereas a high k value creates a sharp, step-like transition.

For Lyme borreliosis, they were x0 = 70 and k = 0.15.

The calculated exposure to CCHFV is

$$CCHFvE={CCHFV}_{th}\times HumanRP\times DenLives\times PresWild$$

where:

CCHFvE: CCHFV exposure.

CCHFVth: CCHFV hazard (the average of suitability for the tick vectors of *Orhtonairovirus hemorrhagiae*).

HumanRP: Density of human rural population.

DenLives: Density of livestock population.

PresWild: Average of the presence/absence (binary) of large ruminants feeding the vector ticks, resulting in a decimal number in the range 0–1.

Normalized as before.

### 2.5. Calculation of Vulnerability

Vulnerability is a complex dimension to implement within high-resolution spatial frameworks due to the intrinsic scales at which socio-economic data are collected [33,34,35,36]. However, establishing a vulnerability index for tick-borne diseases remains essential for ongoing and future risk monitoring. Vulnerability to tick-borne hazards cannot be evaluated using metrics developed for other arthropod vectors (such as mosquitoes), because tick management does not share national vector-control infrastructures or unified municipal intervention techniques. While existing frameworks evaluate national capacities to manage mosquito-borne zoonoses equivalent baseline metrics for ticks lack comparable reliability. Furthermore, the current literature regarding vulnerability to tick-borne pathogens relies primarily on descriptive mapping rather than dynamic indices applicable across uniform administrative levels.

To resolve this limitation, we developed a standardized vulnerability index utilizing country-level operational metrics that account among others for: travel time to health resources [35], the Human Development Index (HDI), physical exposure to zoonoses, life expectancy at birth, expected years of schooling, or the economic dependency index [36]. These indicators represent robust proxies for a population’s quality of life, healthcare accessibility, economic resilience, and sanitary resource governance, sourced from validated international monitoring repositories [34].

The calculated vulnerability is

VUL=LDE×Exp×TThr×Pd×GNI×(1−HDI)×(100−LE)×(100−YS)×EDI

where:

VUL: Vulnerability.

LDE: Percent of population exposed to *Borrelia burgdorferi* s.l. (replaced by Percent of population exposed to CCHFv if calculating vulnerability by CCHFV).

Exp: Physical exposure to zoonoses. This index is obtained from the INFORM report and is a generalist measure of the exposure of the population to zoonoses of any type.

TThr: Travel time to health resources. It is calculated from the two raster layers displaying access time to health resources, both walking and motor times [35], as the square root of Walking time + Motor time, divided by 100.

Pd: Total human rural population in the country.

GNI: Density of livestock population, which is the sum of the density of cattle, goats, and sheep.

HDI: Human Development Index, obtained from the INFORM report.

LE: Life expectancy at birth [34].

YS: Expected years of schooling [34].

EDI: Economic dependency index [34].

Protective factors (e.g., Human Development Index, Life Expectancy, Physicians Density, Expected Years of Schooling) were inverted prior to indexing (100 − X or 1 − X) so that higher rescaled values uniformly denote higher vulnerability/lower adaptive capacity. All the intermediate values above were normalized to 0–100 before entered into the calculation of VUL, using a sigmoid function with the sigmoid parameters as described before. In this case, for each value, x0 was the median of the distribution of each series of values, and k = 0.15. Each sub-indicator was rescaled into a 0–100 continuous bounded range using the median-centered sigmoid function (x0 = median, k = 2.15).

The Human Development Index (HDI) is a composite statistical measure of life expectancy, education, and per capita income indicators sourced from the INFORM risk framework [34] (available at https://drmkc.jrc.ec.europa.eu, accessed on 1 December 2025). The economic dependency index is a structural ratio calculated within the INFORM report that balances demographic distributions with age-specific economic behaviours, such as labour market participation, consumption patterns, and public transfers [36].

### 2.6. Validation of the Models

A rigorous, multi-tiered validation protocol was implemented to assess the predictive performance of the spatial models. The validation workflow was structured into three distinct stages corresponding to: (1) individual species distribution models for human-biting ticks, (2) the *Borrelia burgdorferi* s.l. exposure index, and (3) the Crimean–Congo haemorrhagic fever virus (CCHFV) exposure index.

#### 2.6.1. Validation of Tick Hazard Models

The spatial accuracy of individual tick suitability models, which constitute the baseline layer for the global Tick Hazard Index, was evaluated across both continuous and binarized predictions. To convert continuous relative habitat suitability surfaces into binary presence–absence maps, an optimal classification threshold was selected by maximizing the True Skill Statistic (TSS=Sensitivity+Specificity−1). This approach minimizes both omission (false negative) and commission (false positive) errors, preventing artificial suitability inflation in geographically under-sampled regions. Model performance was systematically quantified using a suite of complementary metrics: sensitivity (measuring the proportion of correctly classified observed presences), specificity (monitoring the correct classification of background points/absences), omission rates (to ensure models do not underestimate territories susceptible to tick establishment), and TSS as a prevalence-independent metric of overall accuracy.

#### 2.6.2. Validation of Pathogen Exposure Models

Direct global validation of pathogen exposure models using field-measured vector infection rates is logistically unfeasible due to the severe scarcity, spatial fragmentation, and temporal heterogeneity of empirical questing-tick survey data worldwide. Consequently, exposure indices were validated against independent, macro-scale epidemiological benchmarks that reflect the spatial footprints of pathogen circulation:

- Lyme Borreliosis: Empirical human case notification datasets compiled between 2013 and 2024 from the US Centers for Disease Control and Prevention (CDC) and European surveillance registries [37,38,39,40]. While case reporting is mandatory in the United States, European surveillance exhibits national heterogeneity, with Lyme neuroborreliosis serving as the primary standardized communicable disease target across European Union member states since 2018.
  Empirical human case notification datasets compiled between 2013 and 2024 were utilized to validate the Borrelia burgdorferi s.l. exposure model. For North America, state-level annual reported case counts and incidence data were retrieved from the US Centers for Disease Control and Prevention (CDC) National Notifiable Diseases Surveillance System (NNDSS, available at https://www.cdc.gov/lyme/datasurveillance/, accessed on 1 December 2025). For Europe, validation data were derived from standardized national surveillance registries and comprehensive published synthesis studies reporting country- and regional-level incidence [39].
  To align these heterogeneous administrative reporting sources with the continuous ecological modeling framework, raw notifications were georeferenced. The sample size for validation comprised all administrative units with continuous annual surveillance reporting between 2013 and 2024 (US states, *n* = 50; European surveillance units/countries, *n* = 31). These observational data were extracted as spatial zonal means at cell centroids to serve as independent out-of-bag validation points against model predictions.
- CCHFV: Human and animal (livestock and large wild ungulate) seroprevalence surveys retrieved from a comprehensive systematic review of peer-reviewed electronic databases covering literature published up to September 2024 [29]. Validation of the CCHFV exposure models was conducted using an independent, open-access dataset previously published and archived on Figshare [29]; the dataset is available at https://doi.org/10.1371/journal.pntd.0013783.s002, accessed on 1 December 2025). The dataset encompasses a total of n=1196 georeferenced observational entries across endemic and at-risk regions in Africa, Europe, and Asia. This includes n=895 animal seroprevalence records spanning 60 countries (covering major livestock hosts such as cattle, sheep, and goats) and n=301 human serological and case surveillance records spanning 39 countries.
  The spatial resolution of the underlying surveillance data is structured according to standardized Global Administrative Areas (GADM), ranging from country-level reporting (GADM Level 0) down to specific district/municipality boundaries (GADM Level 3). For integration into the validation pipeline, observational data were georeferenced to their finest reported administrative centroids or zonal boundaries and extracted onto the model’s regular spatial grid domain to calculate out-of-sample performance metrics.

To quantify how effectively the multi-trophic exposure models predict these empirical epidemiological targets without introducing spatial autocorrelation or data leakage, an out-of-bag validation scheme was implemented within a spatial block Random Forest framework. To evaluate model performance while accounting for spatial autocorrelation, a spatially block-stratified k-fold cross-validation scheme (k = 5) was implemented. In this framework, spatial blocks were held out sequentially during model training. Final validation metrics, including Sensitivity, Specificity, True Skill Statistic (TSS), and overall Accuracy, were calculated strictly by pooling the predictions across these spatially independent holdout folds. Internal out-of-bag (OOB) error estimates derived during individual Random Forest model iterations were restricted to hyperparameter tuning and were not used to compute the final reported validation statistics.

Raw maps were obtained from https://www.gadm.org/data.html (accessed on 1 December 2025) for both the GADM3 regions and the countries. The maps are freely available for academic use and other non-commercial use. Redistribution, or commercial use is not allowed without prior permission. The maps were projected and modified using open source software, namely qGIS (v 3.44.7, available at https://qgis.org) and Scimago Graphica (v 1.0.55, available https://www.graphica.app/, accessed on 1 December 2025). See Table 1 for a summart of methodological approaches.

## 3. Results

### 3.1. Validation of the Models

Model validation for the 40 tick species yielded high predictive accuracy across independent spatial evaluation blocks (mean Continuous Boyce Index = 0.988, Omission Rate = 0.220, Sensitivity = 0.780, Specificity = 0.863, TSS = 0.643; see Table S1 for species-specific confusion matrices). For the *Borrelia burgdorferi* s.l. exposure model, out-of-bag validation against independent empirical notification datasets yielded a Sensitivity of 0.91 and a Specificity of 0.96, resulting in a True Skill Statistic (TSS) of 0.87, an overall accuracy of 0.94, a Root Mean Squared Error (RMSE) of 0.1044, and Cohen’s kappa = 0.87. For the CCHFV exposure model, validation against independent seroprevalence benchmarks yielded a Sensitivity of 0.82 and a Specificity of 0.87, resulting in a TSS of 0.69 (calculated as 0.82 + 0.87 − 1 = 0.69), an overall accuracy of 0.84, an RMSE of 0.1434, and Cohen’s kappa = 0.67. All model metrics reflect robust discrimination of eco-environmental hazard and population exposure.

### 3.2. Global Patterns of Tick Environmental Suitability and Population Exposure

**Figure 1:**
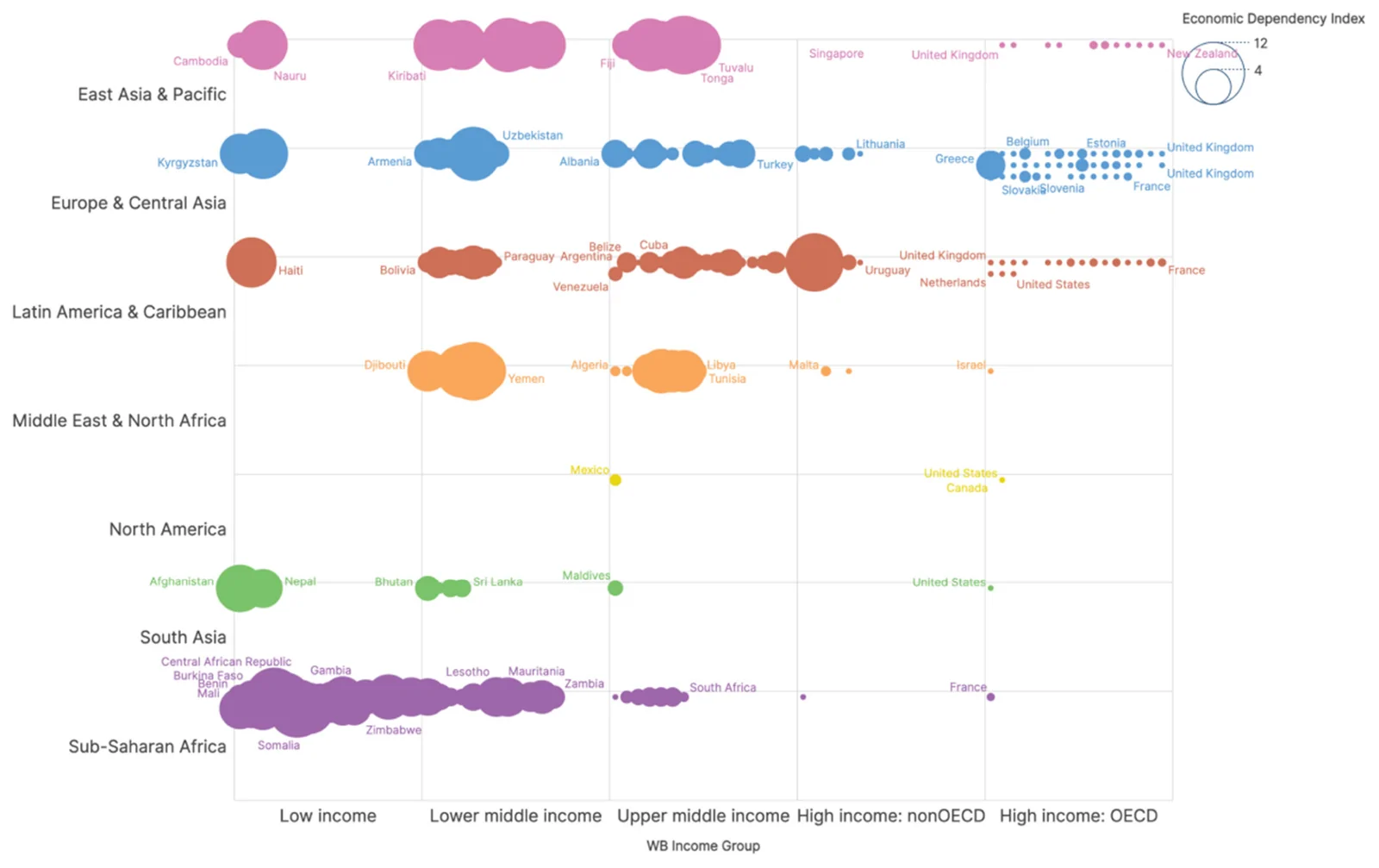
Classification of countries and territories according to their economic dependency index (EDI), categorized following World Bank regions and income levels. Color represents the region; the size of each circle is proportional to the EDI. The chart is intended as an appraisal of country categories rather than an assessment of individual territories. This index reflects environmental suitability independently of population size, economic, or health status, depending on tick distribution. Most countries in the Mediterranean region, Central Asia, eastern China, and eastern North America exhibit the highest index values. Tick presence on humans is predominant across large territories of northern, central, and southern Africa.

The global baseline analysis encompassed 250 countries and territories classified under the World Bank regional framework, capturing distinct macroeconomic dependencies and baseline income contexts (Figure 1).

**Figure 2:**
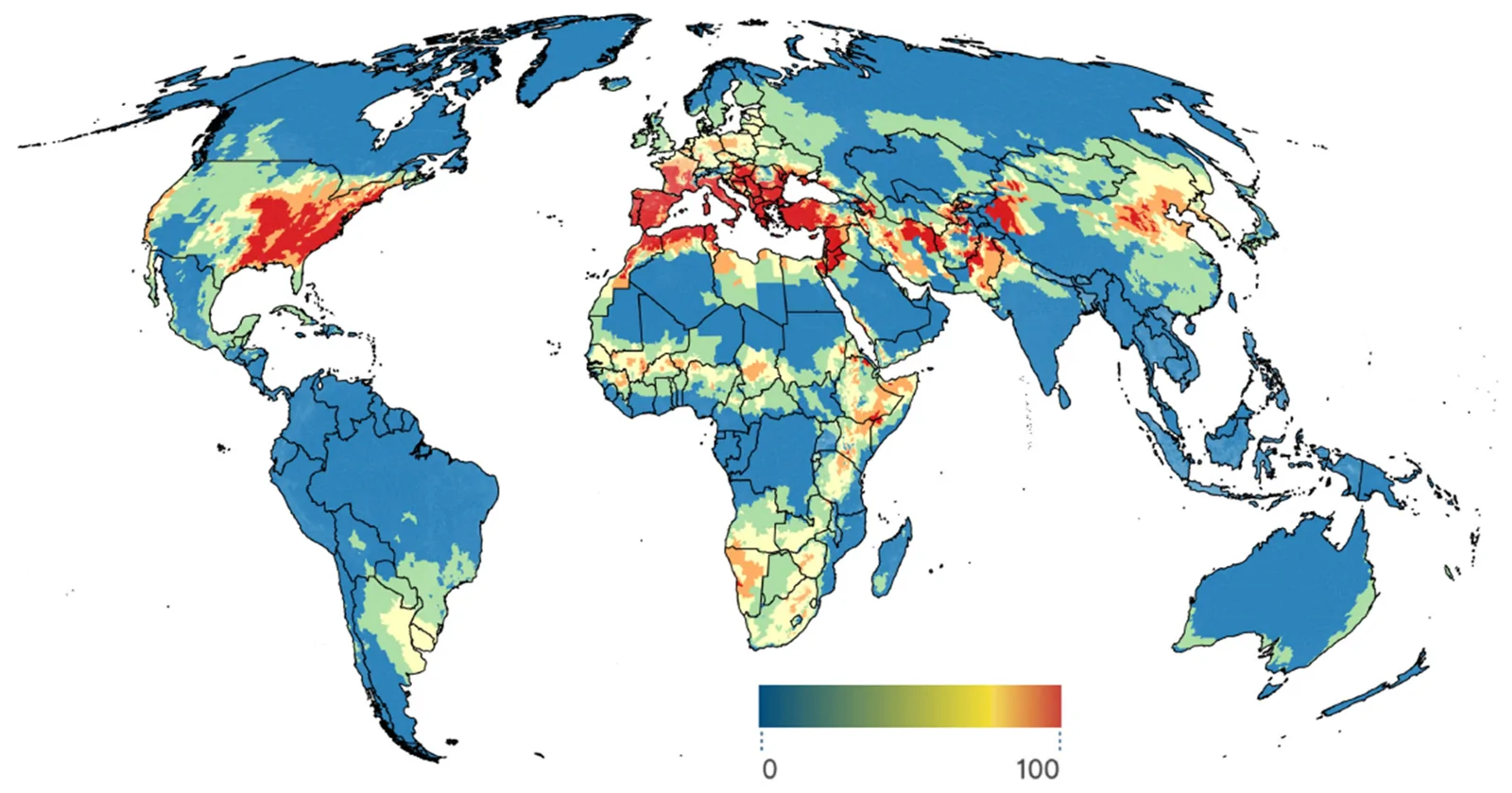
Global high-resolution distribution of the Tick Hazard Index at the GADM level. Values are plotted according to the smallest administrative divisions mapped in the study to avoid distortions caused by country size or non-contiguous territories. Values are rescaled to a 0–100 relative index.

At a global scale, the Tick Hazard Index (THI), representing baseline environmental suitability and tick presence, reveals a highly structured, heterogeneous pattern of biological hazard rather than a uniform latitudinal gradient (Figure 2). Tick hazard clusters into well-defined bioclimatic hotspots across the Northern Hemisphere, specifically concentrated in eastern North America, southern Canada, the Mediterranean basin, the Near East, Central Asia, and temperate East Asia (including China and the Korean Peninsula). Conversely, the Afrotropical realm displays a highly fragmented configuration, characterized by discontinuous hotspots extending along sub-Saharan tracks, the Rift Valley, and into southern Africa, a distribution pointing to strict continental-scale eco-climatic constraints. Complete set of data is available in Table S2.

Central tendencies of the THI varied markedly among world regions. The highest median environmental suitability values were found in Europe and Central Asia (56.1; Q1: 36.7; Q3: 73.1), North America (51.4; Q1: 24.2; Q3: 65), and the Middle East and North Africa (49.7; Q1: 27.7; Q3: 72.3). Substantial but secondary baseline values were recorded across South Asia, Sub-Saharan Africa, East Asia and Pacific, and Latin America & the Caribbean.

**Figure 3:**
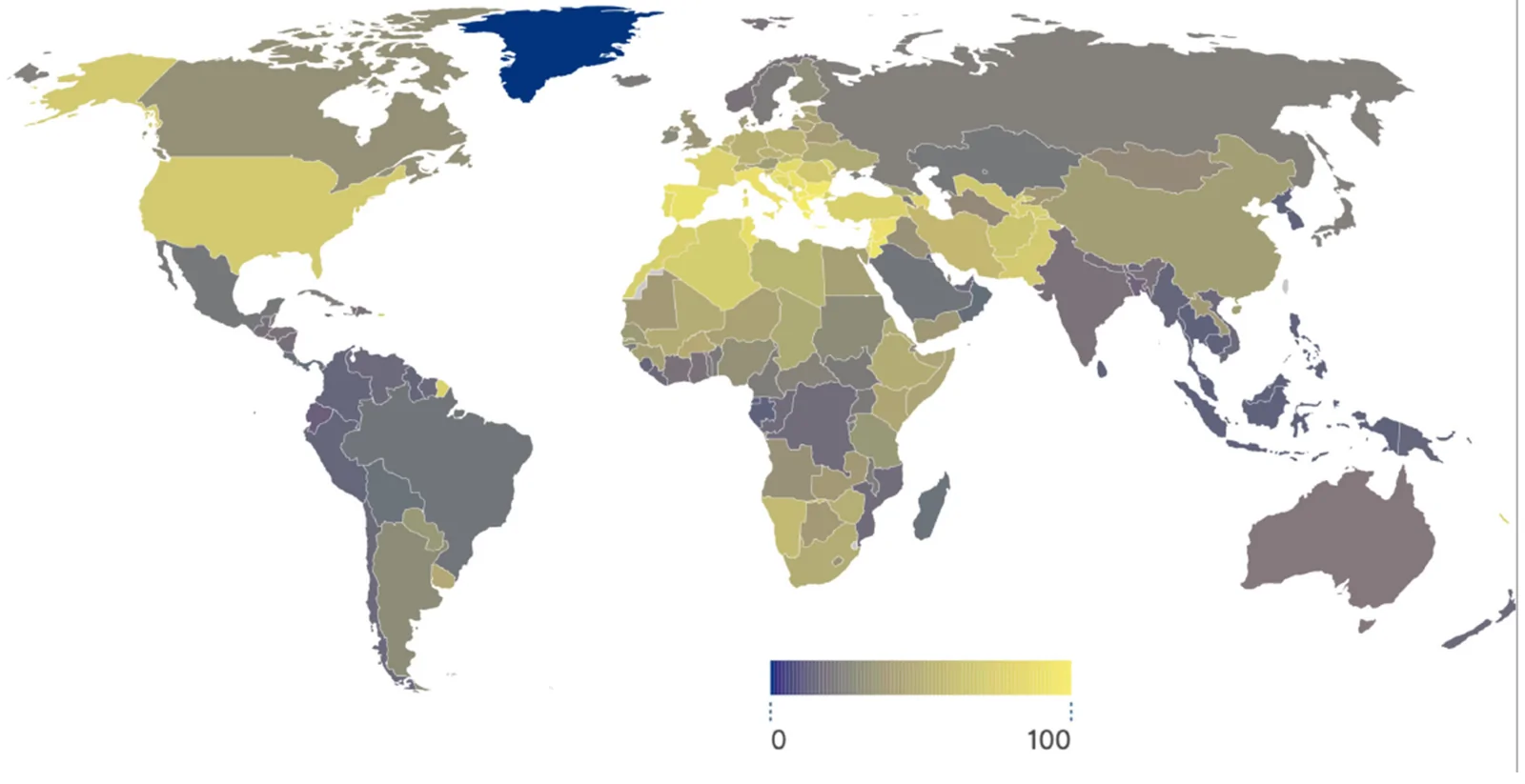
World population exposure by ticks. Values are plotted according to the rural population in the GADM3 administrative divisions of each country, weighted by the tick hazard index of each territory (as in Figure 1), and then averaged at the national level.

When environmental suitability is weighted by human and livestock demographics to derive population exposure, virtually all inhabited territories exhibit non-zero values, although the magnitude shifts significantly across borders (Figure 3). Incorporating these anthropogenic modulators effectively smooths localized micro-climatic hotspots and accentuates macro-regional boundaries. Notably, cross-comparing high-resolution suitability surfaces with aggregated exposure maps highlights how national-level data scaling inevitably obscures fine-scale, sub-national spatial heterogeneity (Figure 2 and Figure 3).

### 3.3. Pathogen-Specific Exposure and Biogeographical Segregation

**Figure 4:**
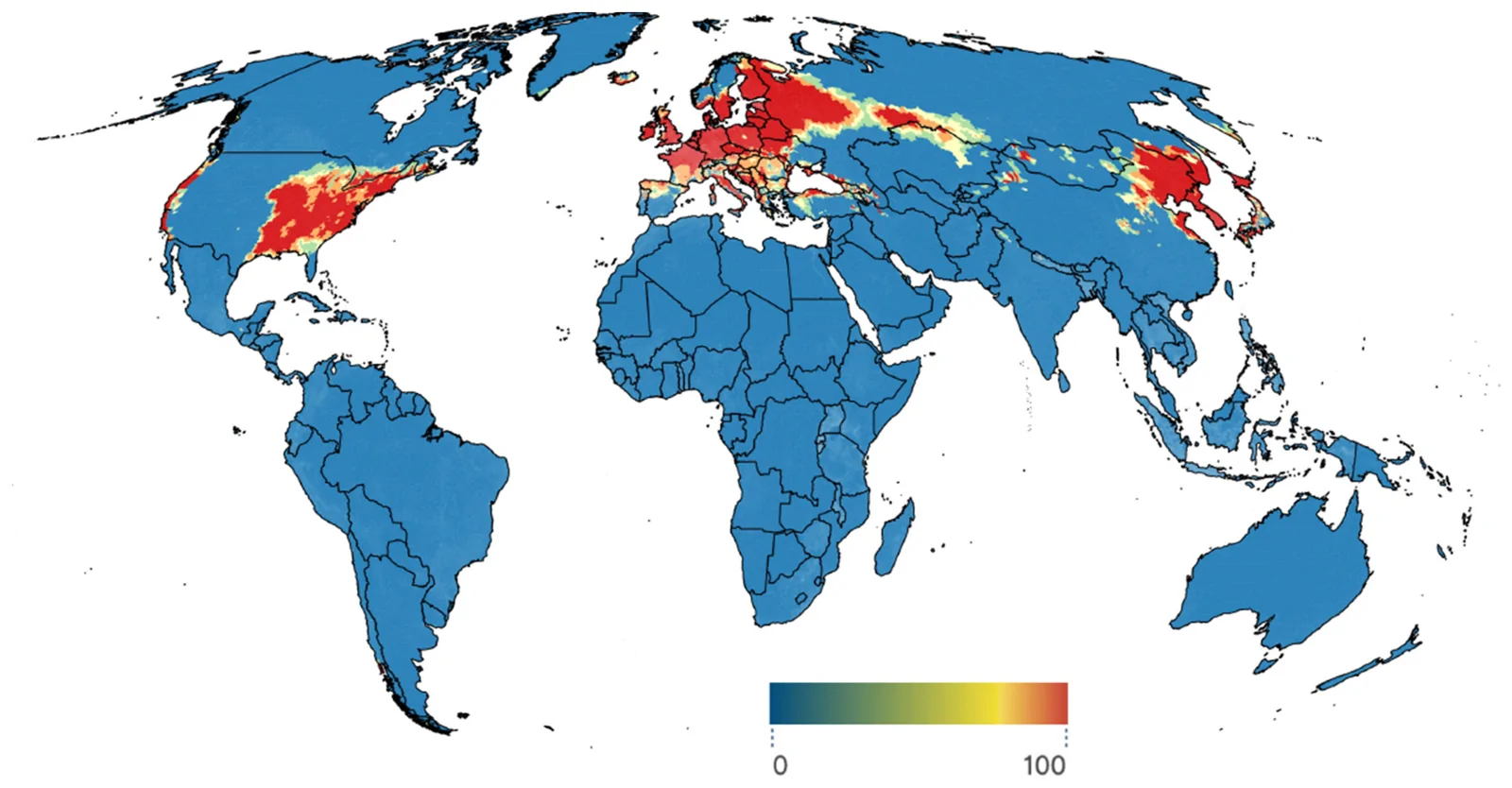
Global high-resolution distribution of the *Borrelia burgdorferi* s.l. (Bb) hazard index at the GADM level. Values were obtained as the tick hazard index for species confirmed as vectors of the pathogen, weighted by the presence of host reservoirs and the large vertebrates that feed substantial numbers of adult ticks.

The pathogen-specific hazard and exposure indices demonstrate strong allopatric structuring and ecological segregation. The *Borrelia burgdorferi* s.l. (Bb) hazard index is almost exclusively restricted to the Holarctic, tightly tracking the overlapping distributions of competent *Ixodes* vectors and their corresponding avian and mammalian reservoir communities (Figure 4). High-hazard corridors extend across southern Canada, northern and central Europe (with pronounced intensity in Scandinavia and Finland), and form a continuous latitudinal belt across the Russian Federation into northern China, the Korean Peninsula, and Japan. The absence of positive Bb hazard values in the Southern Hemisphere reflects the ecological boundaries governing this transmission system, and the probable absence of known vectors in the region.

**Figure 5:**
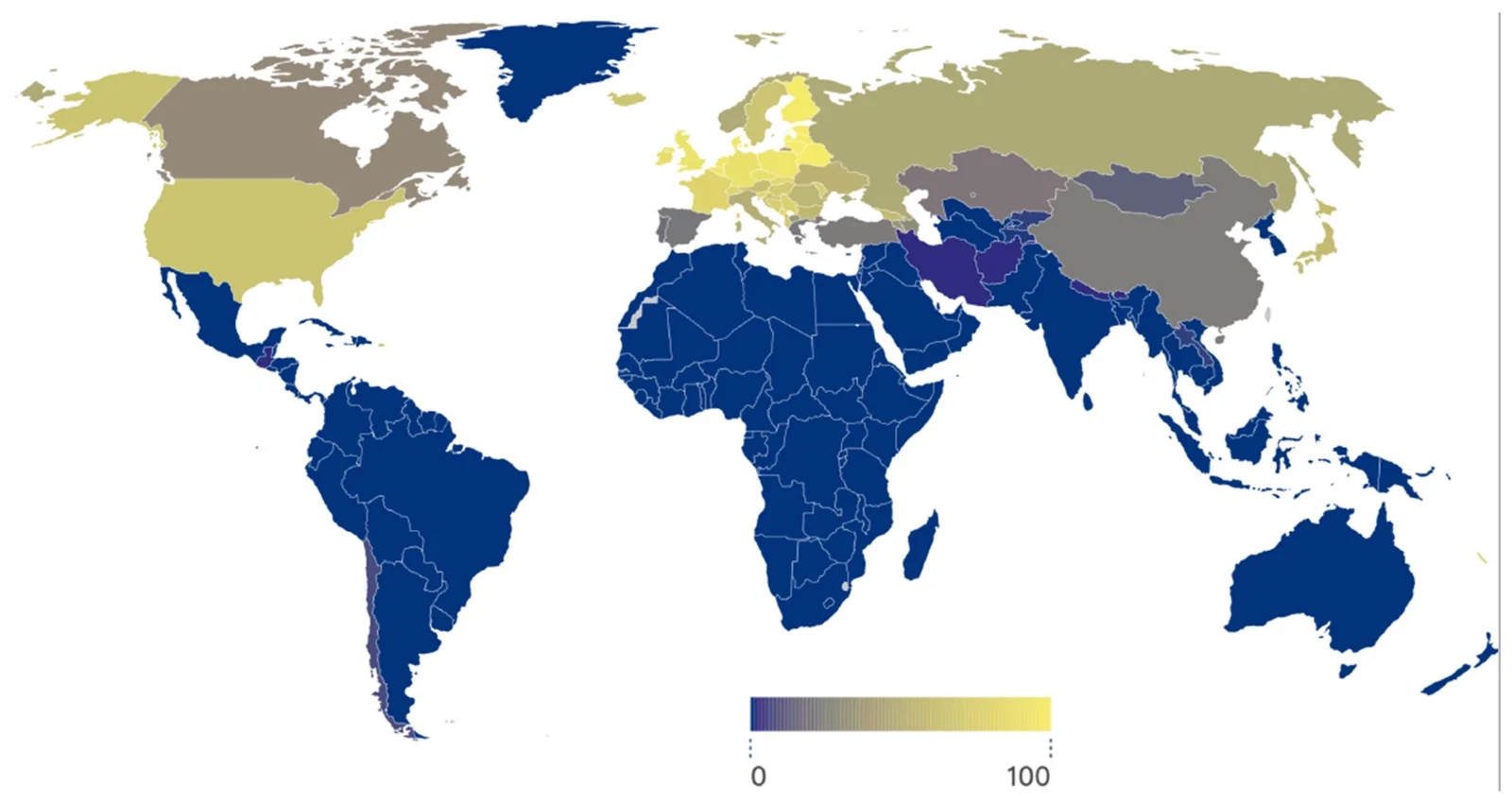
World population under risk by *Borrelia burgdorferi* s.l. Values are plotted according to the rural population in the smallest geographical territories of each country, weighted by the tick index of each territory (as in Figure 3), and then averaged at the national level. Territories belonging to the same country (such as islands in the Caribbean or Pacific Ocean) were disaggregated from the metropolis to avoid biased calculations.

This strict geographic partitioning remains evident when evaluating human exposure metrics (Figure 5). Exposed population potential is heavily concentrated in Europe and Central Asia, which emerged as the primary global hotspot (median population exposure: 68.4%; Q1: 55.4; Q3: 92.8), followed by North America (45.4%; Q1: 19.3; Q3: 58.5). Exposure values across all remaining global regions were negligible, positioning Lyme borreliosis eco-environmental suitability predominantly as a Northern Hemisphere concern with an asymmetric burden on European territories.

**Figure 6:**
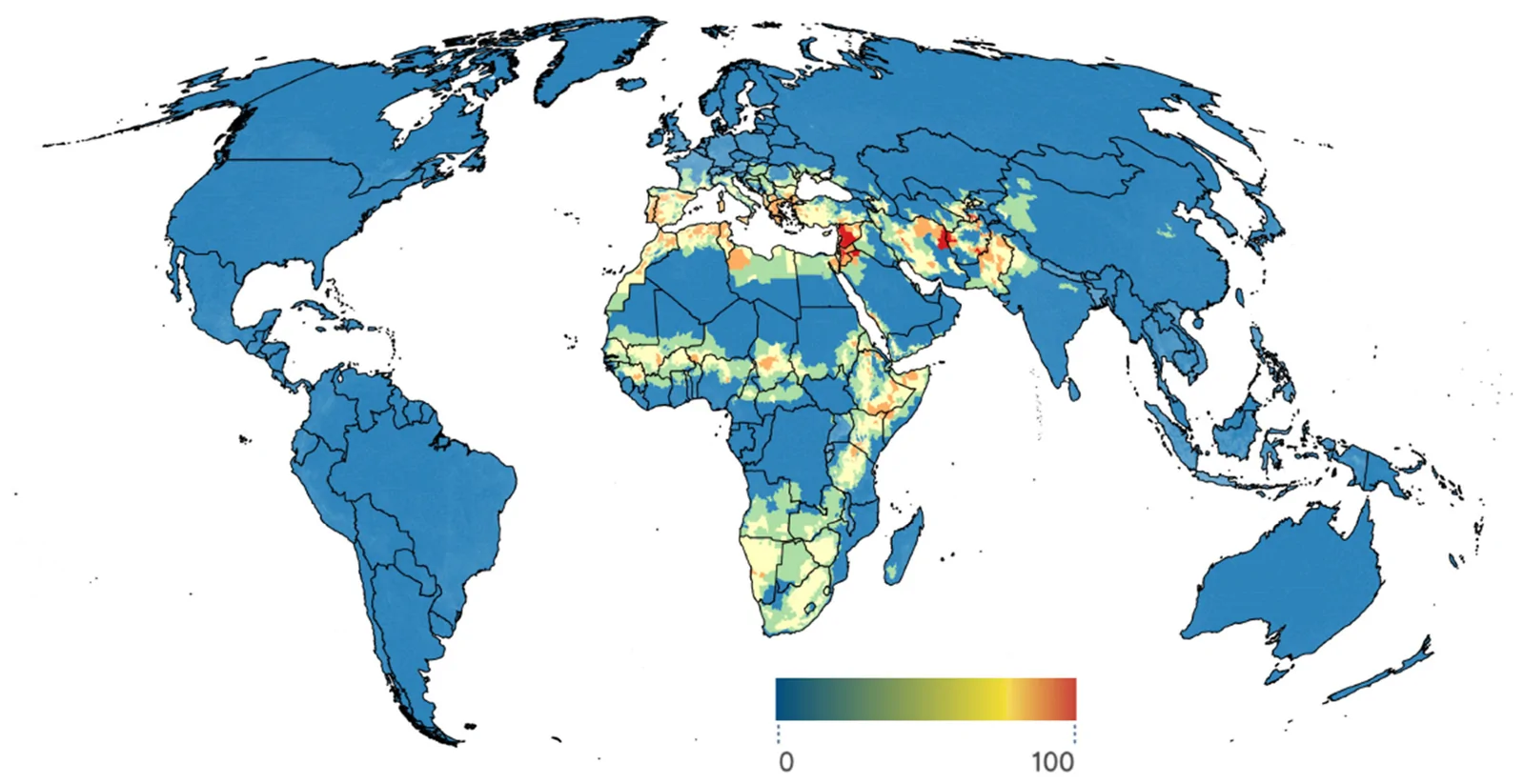
Global high-resolution distribution of the Crimean–Congo haemorrhagic fever virus (CCHFV) hazard index at the GADM level. Values were obtained as the tick hazard index for species known to act as vectors of the pathogen, weighted by the presence of large vertebrates feeding adult ticks. In the case of CCHFV, ticks act as both vectors and reservoirs; thus, separate vertebrate reservoirs were not included in the primary weightings.

In contrast, the CCHFV Hazard Index displays an almost complementary, Old World–restricted spatial template (Figure 6). Environmental hazard for CCHFV is concentrated across the Palearctic and Afrotropical regions, matching the ecological niches of *Hyalomma* ticks and their trophic associations with livestock and wild ungulates. High-hazard landscapes span from the Mediterranean basin through the Middle East into Central Asia, alongside expansive suitable zones in Sub-Saharan Africa (the Rift Valley and southern African savannas). Characteristically, the CCHFV hazard index is completely absent from the Americas.

**Figure 7:**
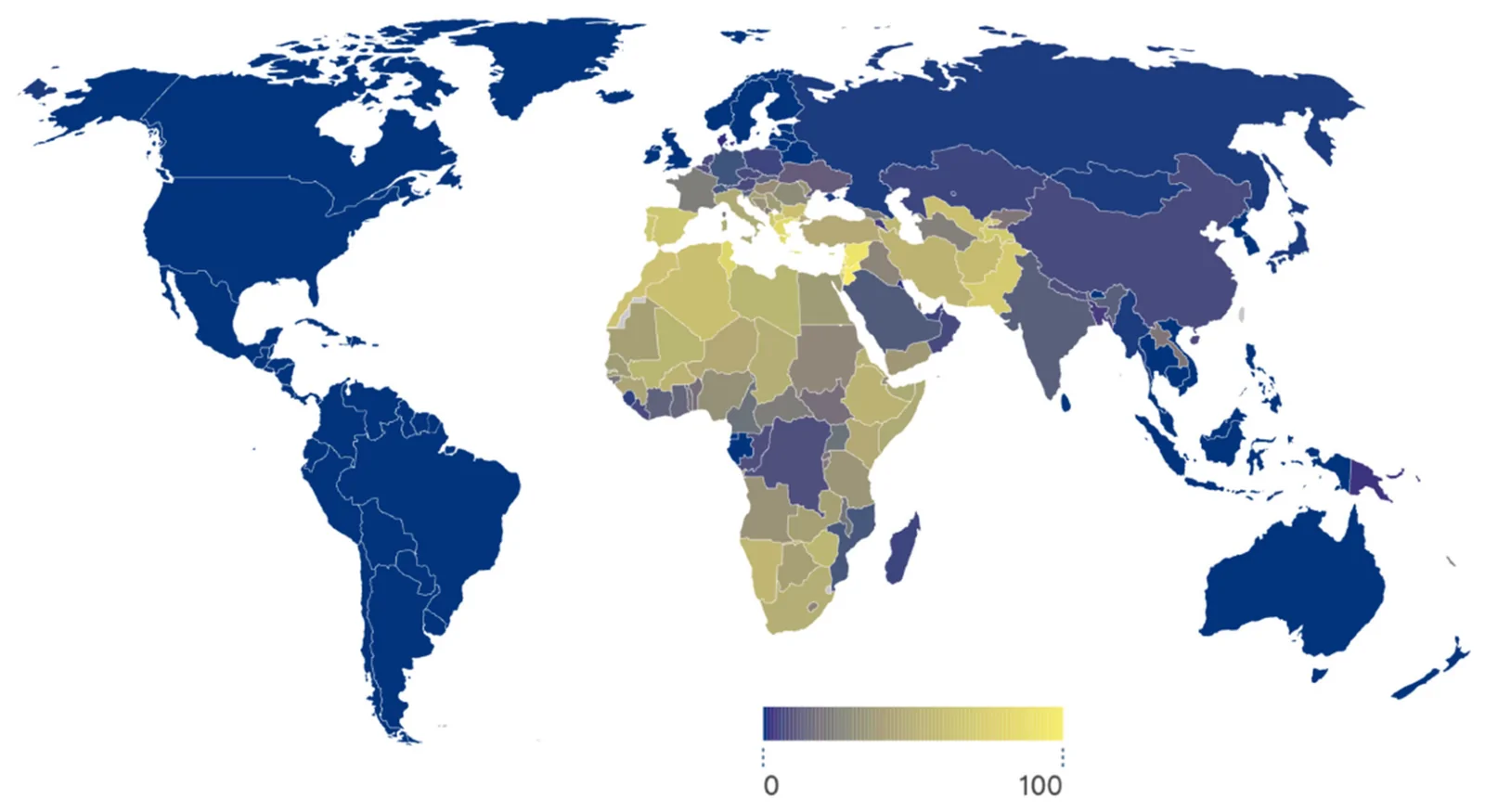
World population exposure to Crimean–Congo haemorrhagic fever virus (CCHFV). Values are plotted according to the rural population in the smallest geographical territories of each country, weighted by the tick index of each territory, and then averaged at the national level. Territories belonging to the same country (such as islands in the Caribbean or Pacific Ocean) were disaggregated from the metropolis to avoid biased calculations. The complete dataset on exposure and vulnerability at the World level is included in Table S3.

Quantifying human and livestock exposure to CCHFV reinforces this strict geographic segregation (Figure 7). The highest systemic population exposure occurs in the Middle East and North Africa (median: 56.1%; Q1: 48.1; Q3: 65.4) and Sub-Saharan Africa (median: 45.0%; Q1: 41.7; Q3: 56.1), while Europe and Central Asia exhibits intermediate, highly variable values (median: 21.1%; Q1: 0.0; Q3: 50.9). Measurable exposure in the rest of the world is non-existent.

### 3.4. Macro-Regional Synthesis and Vulnerability Convergence

**Figure 8:**
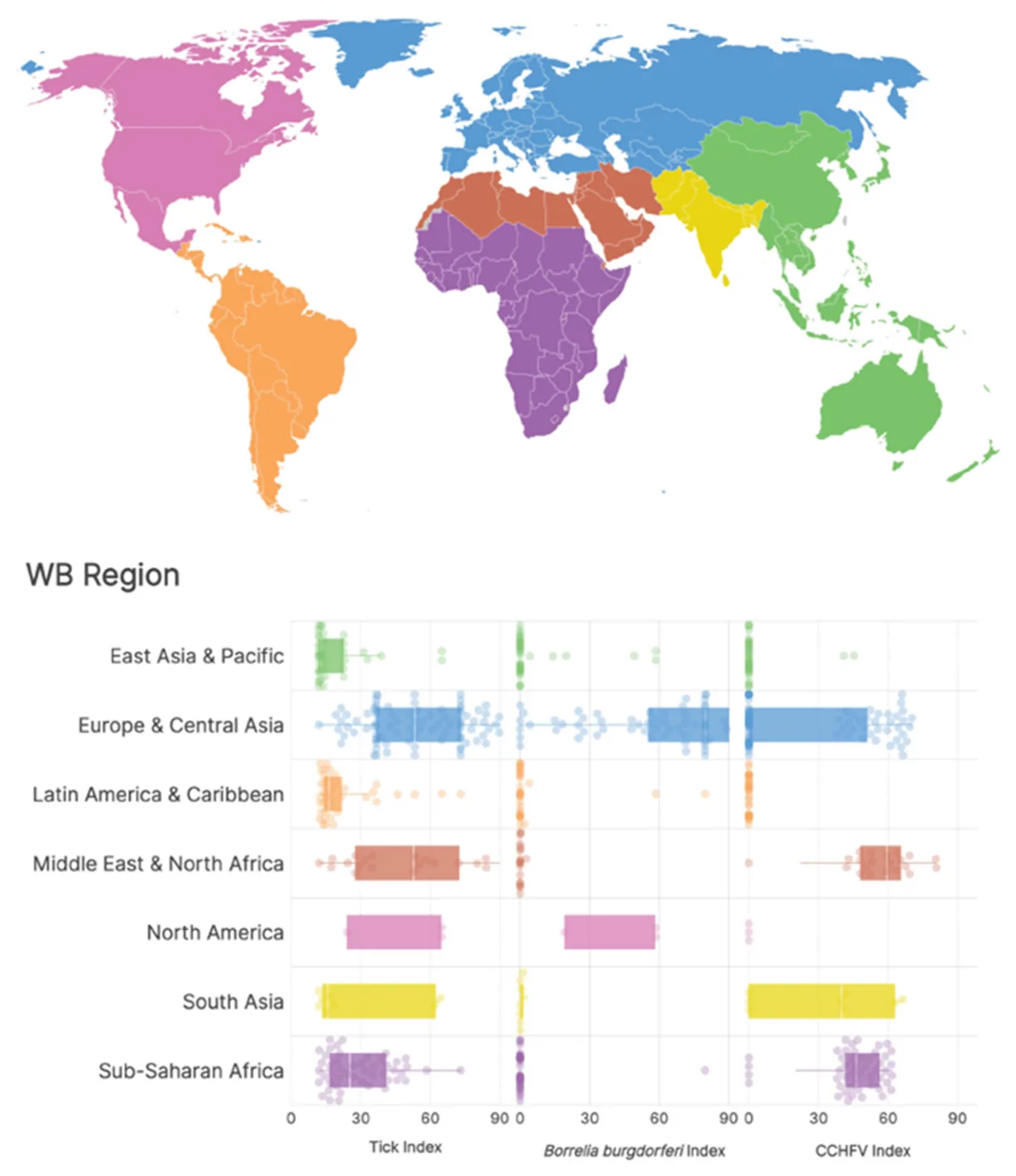
Values of indices for each country grouped by territory. Countries were grouped according to World Bank geographical divisions. The chart shows the value of each index for each country. Notably, Europe, Central Asia, and the Middle East and North Africa exhibit the highest Tick Index values, with the largest variation in Europe and Central Asia reaching nearly 90%. Conversely, the Bb index shows positive values only in Europe, Central Asia, and North America, with significant variation across these regions. The CCHFV index shows positive values concentrated in Europe, Central Asia, and Sub-Saharan Africa.

**Figure 9:**
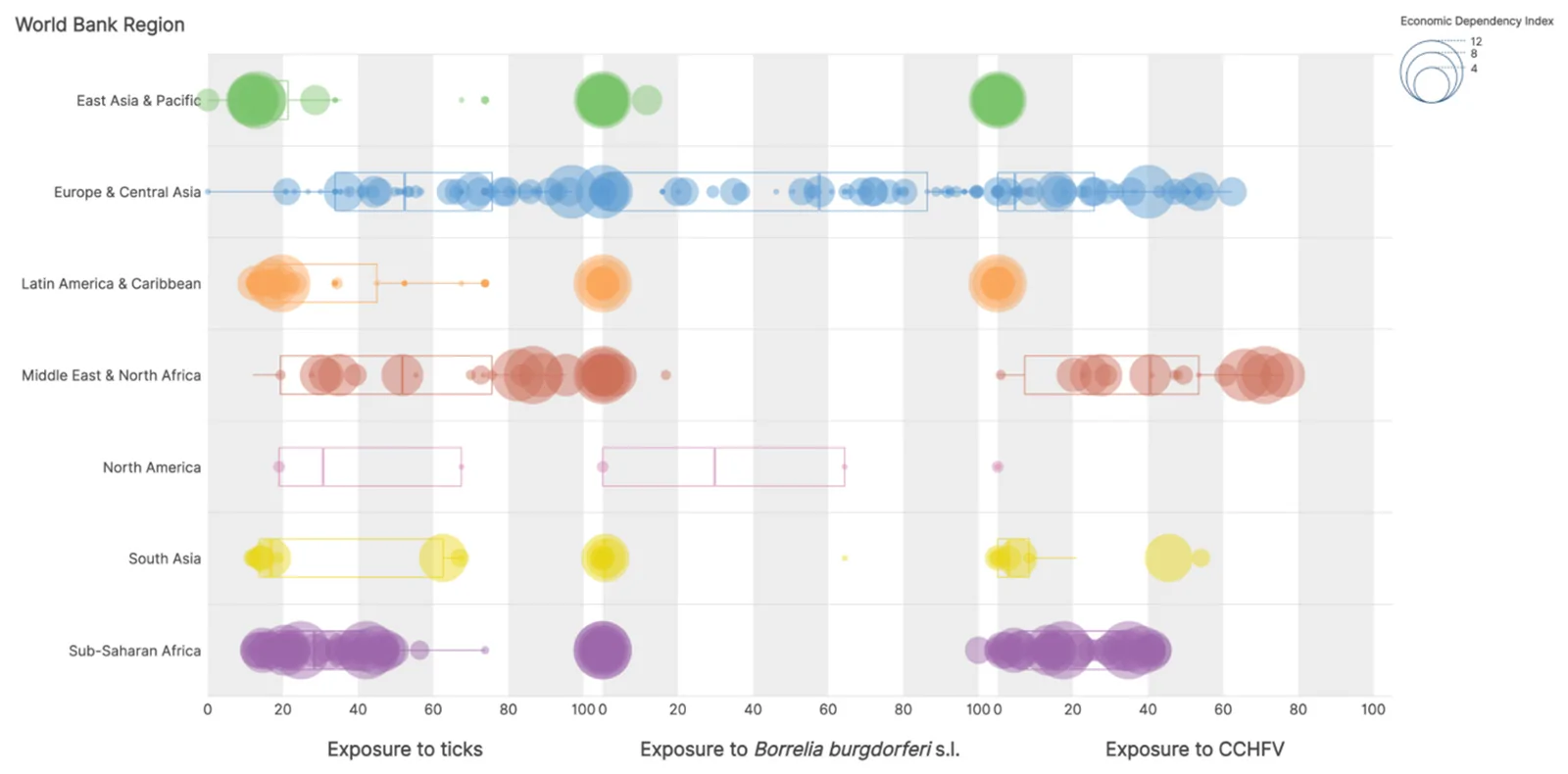
Cross-values of country exposure indices grouped by the economic dependency index. Indices are grouped according to World Bank geographical divisions. The chart illustrates the percentage of exposure to ticks, *Borrelia burgdorferi* s.l., or Crimean–Congo haemorrhagic fever virus, relative to a country’s economic dependency index. Socioeconomic vulnerability is highest in Sub-Saharan Africa and the Middle East and North Africa, while European countries exhibit a wide range of values.

**Figure 10:**
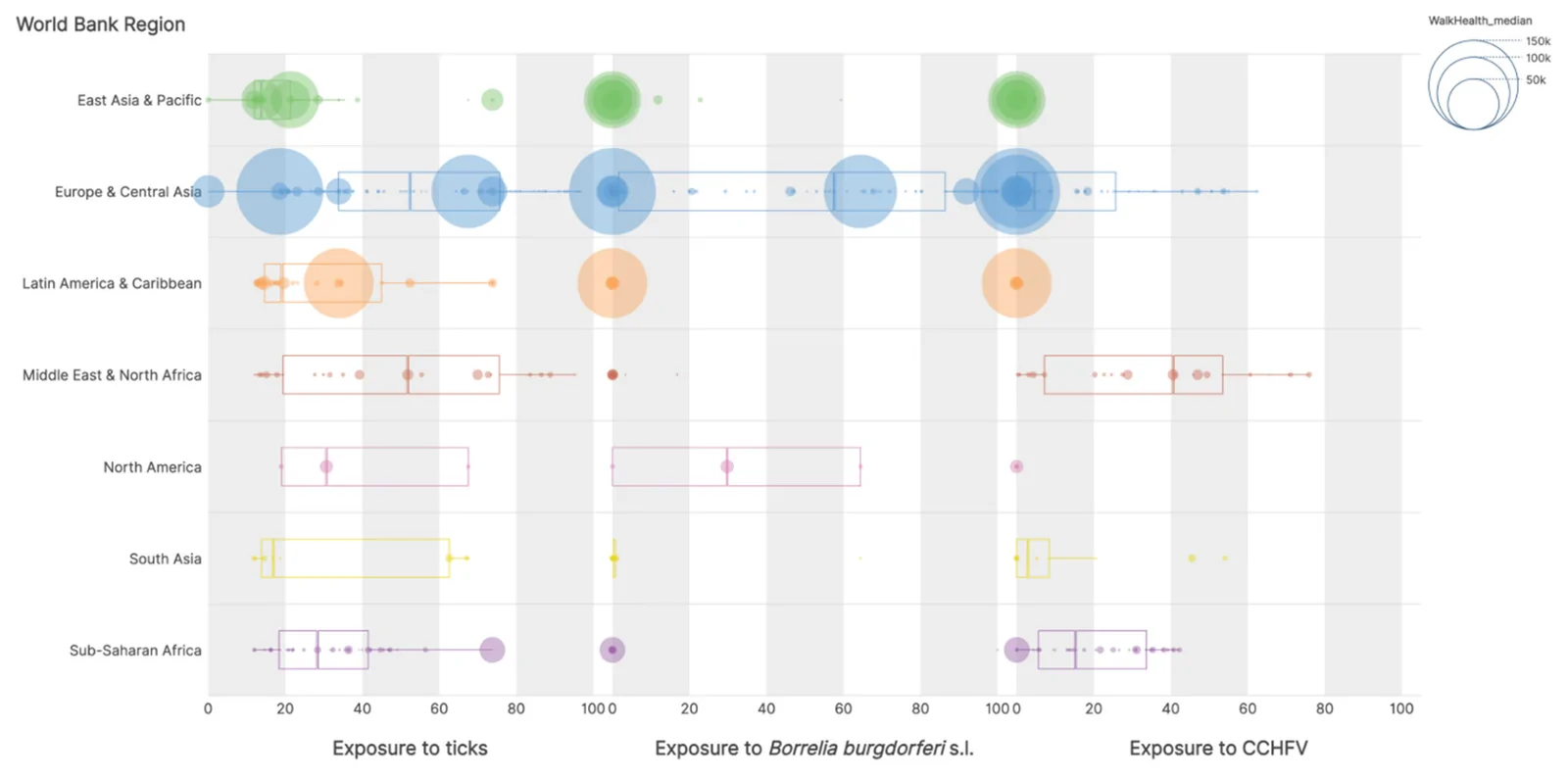
Cross-values of country exposure indices measured by walking distance to health facilities. Indices are grouped according to World Bank geographical divisions. The chart illustrates the percentage of exposure to ticks, *Borrelia burgdorferi* s.l., or Crimean–Congo haemorrhagic fever virus, relative to distance in kilometers to the nearest health facility (motorized arrival time evaluated via a raster friction surface).

**Figure 11:**
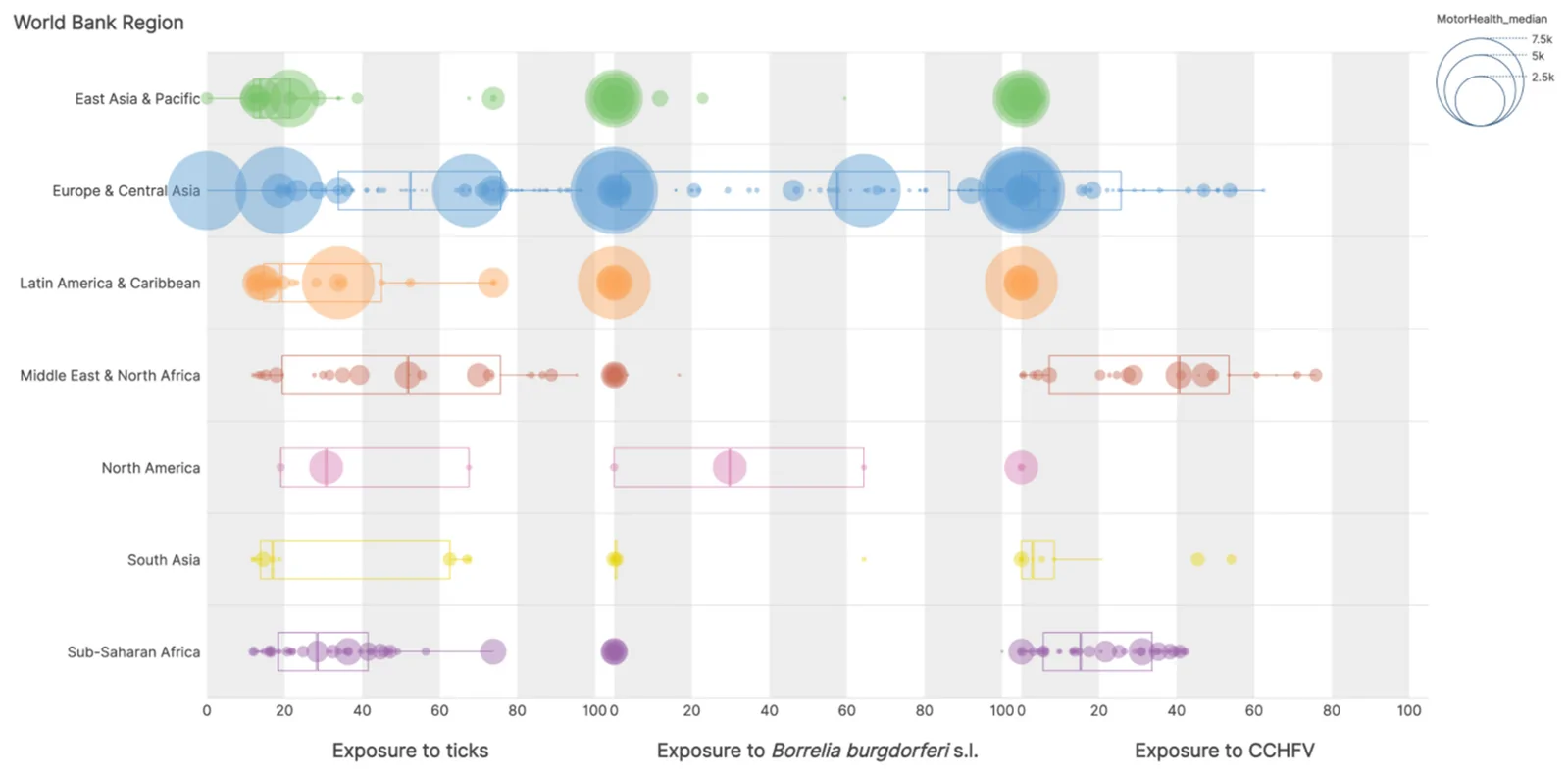
Cross-values of country exposure indices measured by motorized distance to health facilities. Indices are grouped according to World Bank geographical divisions. The chart illustrates the percentage of exposure to ticks, *Borrelia burgdorferi* s.l., or Crimean–Congo haemorrhagic fever virus, relative to distance in kilometers to the nearest health facility (walking arrival time evaluated via a raster friction surface).

A multi-index synthesis underscores the deep evolutionary and ecological divergence between these two pathogen transmission systems (Figure 8, Figure 9, Figure 10 and Figure 11). The generalist tick hazard index reaches its highest and most variable configurations across the continuous landmass of Europe, Central Asia, and North Africa. Furthermore, the individual pathogen exposure profiles split into distinct epidemiological arenas: Bb suitability remains strictly localized to the Nearctic and western/northern Palearctic, whereas CCHFV exposure dominates the Afrotropical and southern Palearctic regions.

Intersecting these spatial exposure metrics with socioeconomic vulnerability indicators revealed a distinct spatial decoupling (Figure 9, Figure 10 and Figure 11). At a global scale, exposure to tick-borne hazards appears independent of national economic dependency ratios and structural healthcare accessibility.

In Sub-Saharan Africa and parts of the Middle East and North Africa, high population exposure directly overlaps with high economic dependency and limited health infrastructure, identifying territories with low systemic resilience to vector-borne pressures. Conversely, Latin America and the Caribbean present a consistently low-exposure profile across all hazard and exposure indices. Collectively, these macro-epidemiological configurations demonstrate a pronounced hemispheric and latitudinal structuring of potential tick-borne disease spatial distributions, where Lyme borreliosis hazards shape the temperate northern landscape and CCHFV hazards define the tropical and semi-arid suitability architectures of the Old World.

## 4. Discussion

### 4.1. Methodological Advances in Spatial Scaling and Ecological Integration

This study establishes a comprehensive macro-ecological framework for quantifying baseline hazard and structural exposure to human-biting ticks and two high-consequence tick-borne pathogens: *Borrelia burgdorferi* s.l. (the etiological agent of Lyme borreliosis) and *Orthonairovirus haemorrhagiae* (commonly designated as Crimean–Congo haemorrhagic fever virus, CCHFV). By executing models at two distinct spatial tiers—high-resolution sub-national administrative units (GADM3) and aggregated country levels—we mapped fine-scale ecological heterogeneities associated with tick-borne transmission processes. Following international risk-assessment terminology (such as the INFORM framework), we define “hazard” as the underlying ecological suitability for vectors and host communities and “exposure” as the spatial overlap between this suitability and human/livestock populations. The framework ensures cross-compatibility with established global risk assessments, providing a standardized, high-resolution global appraisal of potential vector and pathogen suitability.

A primary technical strength of this architecture is its underlying ~4 km spatial resolution. Operating at this grain allows the models to capture critical micro-climatic and landscape variations that are inherently obliterated by coarse, country-level spatial assignments [40]. Modeling vector-borne systems at insufficient resolutions introduces geographical biases, distorting the estimated climate-niche space [41]. For instance, national-scale assessments of Lyme borreliosis (e.g., [42]) inevitably mask sub-national climate gradients that govern vertebrate reservoir densities and vector survival. Mapping tick presence, whether from actual field records or modeling approaches, has commonly relied on raster surfaces that ignore administrative divisions used for public health impact analyses, a frequent practice in Europe [22]. An administrative-level paradigm is more commonly adhered to in North America, where county-level resolutions inform localized public health interventions (e.g., [17]). This approach makes our outputs applicable for regional health authorities.

Furthermore, driving these indices through the joint spatial modeling of vectors, primary vertebrate hosts, and competent reservoirs marks a distinct shift toward community-ecology frameworks [43] that embrace a One Health approach. This approach captures the biological mechanisms underlying potential pathogen persistence, offering a major advantage over classic frameworks that rely exclusively on scattered vector presence records or passive, under-reported human case notifications [38,39,44]. Modeling tick-borne pathogens without integrating reservoir host distributions provides an incomplete ecological picture because persistent transmission cycles cannot be maintained without considering the reservoirs [41,45]. Our approach is radically different since it provides a baseline to identify zones of high potential exposure to *B. burgdorferi* s.l., where the simultaneous presence of competent vectors, host reservoirs, and host-mediated tick population amplification supports suitable conditions for transmission.

### 4.2. Entomological Gold Standards vs. Multi-Dimensional Hazard Constraints

A critical finding of our framework centers on the conceptual and logistical limitations of relying on pathogen prevalence in questing ticks, historically considered an entomological standard, for large-scale spatial mapping. While infection prevalence reflects local, instantaneous biological pressure, it is an imperfect proxy for actual human clinical incidence or individual risk. True public health risk is a multi-dimensional function modulated by human behaviour in the landscape, occupational exposure frequencies, climate impacting tick life cycles, personal protection, and systemic healthcare accessibility [46]. We explicitly avoided attempting to model averaged pathogen prevalence in questing ticks because (a) prevalence fluctuates rapidly across time and space due to complex, fine-scale dynamics that are difficult to parameterize globally, (b) empirically measured prevalence data remain unavailable across the vast majority of the Earth’s surface, and (c) the impact of tick abundance in the exposure [46]. We therefore adopted a broad ecological approach aimed at categorizing areas based on environmental suitability and potential population exposure using established relationships among vectors, reservoirs, and pathogens [27,32,47,48,49,50].

For CCHFV, these challenges are compounded by severe data taphonomy and sampling constraints, resulting in a scarcity of accurate spatial data for viral detections in field populations. Outside hyper-endemic foci, natural CCHFV infection rates in field-collected ticks are highly micro-localized, requiring extensive sampling efforts and substantial logistical overhead to confirm viral presence via RNA detection. Standard sampling methodologies, such as flagging and dragging, exhibit low capture efficiencies for hunting ticks like *Hyalomma* spp., necessitating direct collection from animal hosts [28,31].

Based on these considerations, this study formulates a standardized set of macro-ecological indices aimed at quantifying entomological hazard and population exposure rather than clinical infection rates. Conceived as a proof-of-concept, the indices specific to *B. burgdorferi* s.l. and CCHFV demonstrate the operational feasibility of this macro-ecological framework. By modeling the ecological niches of vectors, key vertebrate hosts, and competent reservoirs together, this framework moves beyond static spatial models. This provides a detailed characterization of the hazard–exposure continuum weighted by human population density, which improves the epidemiological relevance of the outputs.

### 4.3. Data Gaps, Scale Mismatch, and Future Horizons

Despite its strong predictive performance, this framework possesses intrinsic structural characteristics that require careful interpretation. The derived indices represent continuous surfaces of eco-environmental hazard, host co-occurrence, and structural population exposure, rather than direct empirical projections of clinical disease cases or individual infection risk. A formal empirical correlation between human case rates and our ecological indices remains an important direction for future research. However, clinical case rates are fundamentally driven by local human-tick contact dynamics, personal behaviors, and reporting practices, and cannot be derived solely from baseline environmental hazard [44].

Similarly, global variance in data availability and quality for wildlife host ranges may represent an ongoing source of uncertainty. We used host distributions as essential proxies for ecological host availability. While spatial modeling bridges data-scarce regions, these surfaces remain probabilistic approximations of transmission suitability based on vector-reservoir co-occurrence and vertebrate host availability for tick population amplification [29,51]. These explanatory variables reflect modeled ecological suitability rather than directly observed infection dynamics.

A prominent operational challenge involved the multi-scale integration of socioeconomic vulnerability indicators. While hazard and exposure operate naturally at high sub-national resolutions (GADM scales), metrics that operate on the calculation of vulnerability are primarily available at national governance scales. We did not downscale national vulnerability statistics to avoid introducing artificial spatial precision into country-level metrics. Instead, aggregating local exposure metrics to match national resolutions represents a pragmatic, top-down strategy to evaluate systemic country-level resilience against localized vector-borne pressures, which is not free of error. Recognized sources of bias are the presence of national territories (e.g., New Caledonia) that are far of the metropolis (e.g., France). The availability of information at the high resolution may be lost or mixed in the process of aggregation of the GADM3 spatial units.

This structural distinction explains why our CCHFV exposure metrics diverge from current national-level INFORM outputs. By expanding the vector array to include confirmed human-biting tick species and modeling their ecological niches at fine spatial grains, our models capture suitable transmission environments that national-scale proxies structurally omit. The global distribution of CCHFV suitability reflects complex ecological interactions among ticks, host vertebrates, and the virus [28,43] and constructing such indices based solely on human case numbers would obscure baseline biological hazard.

## 5. Conclusions

This study introduces a globally harmonized, high-resolution framework that integrates vector distributions, host communities, and human–livestock demographics to quantify environmental hazard and population exposure for major tick-borne zoonoses. By shifting the paradigm from binary distribution masks to multi-trophic suitability gradients at sub-national scales, the developed indices capture fine-scale spatial heterogeneities that are obscured by country-level assessments. The empirical decoupling observed between tick-borne exposure and macroeconomic indicators highlights that biological hazard spans diverse geographic and economic boundaries. However, the spatial overlap of high exposure with limited healthcare infrastructure and high dependency ratios in regions such as Sub-Saharan Africa and parts of the Middle East underscores systemic vulnerabilities to vector-borne pressures.

Importantly, these indices measure ecological suitability for ticks, host co-occurrence, and structural population exposure rather than clinical incidence or individual infection risk. The resulting continuous surfaces offer a flexible, transferable, and dynamically scalable platform for international public health prioritization, cross-border ecological comparisons, and vector-borne disease management. Future iterations incorporating dynamic wildlife host updates and regional vulnerability layers will further refine this tool, bridging community ecology and public health policy.

## Figures and Tables

**Table 1:** Methodological summary of global indices, input predictors, spatial resolutions, formulas, and validation frameworks.

Index/Metric	Primary Input Variables	Spatial Resolution	Transformation and Weighting Rules	Output Range	Validation Dataset
Tick Hazard Index (THI)	40 human-biting tick species occurrence records; 15 TerraClimate harmonic coefficients (TMAX, TMIN, VPD)	4 km (raster) aggregated to GADM3	Random Forest continuous suitability; Geometric mean across present species per GADM unit	0–100 (Relative Index)	Spatial block cross-validation (70% train/30% test).
*Borrelia burgdorferi* s.l. Hazard Index	Suitability of 9 confirmed *Ixodes* vector species (*I. ricinus*, *I. scapularis*, *I. persulcatus*, etc.)	4 km aggregated to GADM3	Geometric mean of confirmed *Ixodes* vector suitability scores (>0)	0–100 (Relative Index)	Overlap with Holarctic vector and reservoir distributions
CCHFV Hazard Index	Suitability of 13 confirmed *Hyalomma* and secondary vector species.	4 km aggregated to GADM3	Geometric mean of confirmed CCHFV vector suitability scores (>0)	0–100 (Relative Index)	Overlap with Old World vector range and ungulate host distribution
Pathogen Exposure Indices	Hazard indices; rural human population density (SEDAC); livestock density (GLEAM); rodent/bird/ungulate reservoir presence (MDD, GAP)	GADM3 administrative unit	Multiplicative weighting normalized via sigmoid logistic curve ($x_{0}=70,k=0.15$)	0–100 (Relative Exposure)	Empirical case notifications (US CDC/Europe) and global serological surveys.
Socioeconomic Vulnerability	8 national metrics (travel time to healthcare, physician density, GNI, HDI, dependency index) from INFORM	Country level (National)	Top-down alignment: local exposure aggregated to national benchmarks	0–100 (Index score)	Sourced from validated international monitoring repositories (INFORM framework)

## Data Availability

All the data generated in this study are available in the Supplementary Material.

## Supplementary Materials

The following supporting information can be downloaded at https://www.mdpi.com/article/10.3390/pathogens15090944/s1, Table S1: Validation data for the models of tick hazard at GADM3 level of spatial resolution. Table S2: Complete set of data of indexes of hazard for ticks. Lyme borreliosis and Crimean–Congo hemorrhagic fever virus, at GADM3 level of spatial resolution. Table S3: Complete set of data at country level of indexes of exposure and vulnerability for tick, Lyme borreliosis and Crimean–Congo hemorrhagic fever virus.

## References

[1] Couret J., Schofield S., Narasimhan S. (2022). The environment, the tick, and the pathogen–It is an ensemble. Front. Cell. Infect. Microbiol..

[2] Gilbert L. (2021). The impacts of climate change on ticks and tick-borne disease risk. Annu. Rev. Entomol..

[3] Nuttall P.A. (2022). Climate change impacts on ticks and tick-borne infections. Folia Microbiol..

[4] García-Carrasco J.M., Crowder D.W., Poh K.C., Mosqueda J., Ueti M.W., Gutierrez-Illan J. (2025). Linking tick and wildlife host distributions to map risk of tick-borne diseases. Parasit. Vectors.

[5] Eisen R.J., Eisen L. (2018). The blacklegged tick, *Ixodes scapularis*: An increasing public health concern. Trends Parasitol..

[6] Semenza J.C., Suk J.E. (2018). Vector-borne diseases and climate change: A European perspective. FEMS Microbiol. Lett..

[7] IPCC (2022). Climate Change 2022: Impacts, Adaptation and Vulnerability.

[8] Rocklöv J., Dubrow R. (2020). Climate change: An enduring challenge for vector-borne disease prevention and control. Nat. Immunol..

[9] Ostfeld R.S., Brunner J.L. (2015). Climate change and *Ixodes* tick-borne diseases of humans. Philos. Trans. R. Soc. Lond. B Biol. Sci..

[10] Randolph S.E. (2004). Evidence that climate change has caused ‘emergence’ of tick-borne diseases in Europe?. Int. J. Med. Microbiol..

[11] Estrada-Peña A., Fernández-Ruiz N. (2022). Is composition of vertebrates an indicator of the prevalence of tick-borne pathogens?. Infect. Ecol. Epidemiol..

[12] Beard C.B., Eisen R.J., Barker C.M., Garofalo J.F., Hahn M., Hayden M., Monaghan A.J., Ogden N.H., Schramm P.J., Crimmins A., Balbus J., Gamble J.L., Beard C.B., Bell J.E., Dodgen D., Eisen R.J., Fann N., Hawkins M.D., Herring S.C. (2016). Chapter 5: Vector-borne diseases. The Impacts of Climate Change on Human Health in the United States: A Scientific Assessment.

[13] Ogden N.H., Lindsay L.R. (2016). Effects of climate and climate change on vectors and vector-borne diseases. Trop. Med. Infect. Dis..

[14] Ebi K.L., Semenza J.C. (2008). Community-based adaptation to the health impacts of climate change. Am. J. Prev. Med..

[15] World Health Organization (WHO) (2021). Guidance on Vulnerability and Adaptation to Climate Change Impacts on Health.

[16] Regier Y., Komma K., Weigel M., Kraiczy P., Laisi A., Pulliainen A.T., Hain T., Kempf V.A.J. (2019). Combination of microbiome analysis and serodiagnostics to assess the risk of pathogen transmission by ticks to humans and animals in central Germany. Parasit. Vectors.

[17] Eisen R.J., Eisen L., Beard C.B. (2016). County-scale distribution of *Ixodes scapularis* and *Ixodes pacificus* (Acari: Ixodidae) in the continental United States. J. Med. Entomol..

[18] Sutherst R.W. (2004). Global change and human vulnerability to vector-borne diseases. Clin. Microbiol. Rev..

[19] Bardosh K.L., Ryan S.J., Ebi K., Welburn S., Singer B. (2017). Addressing vulnerability, building resilience: Community-based adaptation to vector-borne diseases in the context of global change. Infect. Dis. Poverty.

[20] Zarowsky C., Haddad S., Nguyen V.K. (2013). Beyond ‘vulnerable groups’: Contexts and dynamics of vulnerability. Glob. Health Promot..

[21] Guglielmone A.A., Robbins R.G. (2018). Hard Ticks (Acari: Ixodida: Ixodidae) Parasitizing Humans.

[22] Estrada-Peña A., de la Fuente J. (2024). Machine learning algorithms for the evaluation of risk by tick-borne pathogens in Europe. Ann. Med..

[23] Scharlemann J.P., Benz D., Hay S.I., Purse B.V., Tatem A.J., Wint G.W., Rogers D.J. (2008). Global data for ecology and epidemiology: A novel algorithm for temporal Fourier processing MODIS data. PLoS ONE.

[24] Hijmans R., Brown A., Barbosa M. (2026). terra: Spatial Data Analysis.

[25] Zhang Y., Sun Y., Wang J., Wu X., Wang L., Yang X., Li S. (2026). Geographical dataset of questing nymphal density for three key tick vector species of Lyme disease. Sci. Data.

[26] Barbet-Massin M., Jiguet F., Albert C.H., Thuiller W. (2012). Selecting pseudo-absences for species distribution models: How, where and how many?. Methods Ecol. Evol..

[27] Springer A., Lindau A., Fachet-Lehmann K., Kämmer D., Bulling I., Knoll S., Mackenstedt U. (2025). Tick hazard in a central European country: Mapping Europe’s principal tick-borne disease vector across Germany. Ticks Tick Borne Dis..

[28] Gargili A., Estrada-Peña A., Spengler J.R., Lukashev A., Nuttall P.A., Bente D.A. (2017). The role of ticks in the maintenance and transmission of Crimean-Congo hemorrhagic fever virus: A review of published field and laboratory studies. Antivir. Res..

[29] Estrada-Peña A., Carnell O., Wijburg S.R., Holding M., Sprong H. (2026). Crimean–congo haemorrhagic fever virus circulates within broad ecological networks of ticks and vertebrates. PLoS Negl. Trop. Dis..

[30] Hofmeester T.R., Coipan E.C., Van Wieren S.E., Prins H.H.T., Takken W., Sprong H. (2016). Few vertebrate species dominate the *Borrelia burgdorferi* s.l. life cycle. Environ. Res. Lett..

[31] Ilboudo A.K., Oloo S.O., Sircely J., Nijhof A.M., Bett B. (2025). Spatial analysis and risk mapping of Crimean-Congo hemorrhagic fever (CCHF) in Sub-Saharan Africa. Sci. Rep..

[32] Si-moussi S., Thuiller W. (2024). Species Habitat Suitability of European Terrestrial Vertebrates for Contemporary Climate and Land Use (Version 1).

[33] Sun Z.S., Wan E.Y., Agbana Y.L., Zhao H.Q., Yin J.X., Jiang T.G., Kassegne K. (2024). Global One Health index for zoonoses: A performance assessment in 160 countries and territories. iScience.

[34] Marin-Ferrer M., Vernaccini L., Poljansek K. (2017). Index for Risk Management. INFORM Concept and Methodology Report—Version 2017.

[35] Weiss D.J., Nelson A., Vargas-Ruiz C.A., Gligorić K., Bavadekar S., Gabrilovich E., Gething P.W. (2020). Global maps of travel time to healthcare facilities. Nat. Med..

[36] Helmy H.E. (2017). The index of “economic independence”: A new measure of an economy’s ability to survive unilaterally. J. Econ. Issues.

[37] Stark J.H., Pilz A., Jodar L., Moïsi J.C. (2023). The epidemiology of Lyme borreliosis in Europe: An updated review on a growing public health issue. Vector-Borne Zoonotic Dis..

[38] Schwartz A.M., Kugeler K.J., Nelson C.A., Marx G.E., Hinckley A.F. (2021). Use of commercial claims data for evaluating trends in Lyme disease diagnoses, United States, 2010–2018. Emerg. Infect. Dis..

[39] Burn L., Vyse A., Pilz A., Tran T.M.P., Fletcher M.A., Angulo F.J., Stark J.H. (2023). Incidence of Lyme borreliosis in Europe: A systematic review (2005–2020). Vector-Borne Zoonotic Dis..

[40] Dagostin F., Erazo D., Marini G., Da Re D., Tagliapietra V., Avdicova M., Rizzoli A. (2026). Predicting the spatio-temporal risk of human tick-borne encephalitis (TBE) in Europe by combining hazard and exposure drivers. One Health.

[41] Estrada-Peña A., Gray J.S., Kahl O., Lane R.S., Nijhof A.M. (2013). Research on the ecology of ticks and tick-borne pathogens—Methodological principles and caveats. Front. Cell. Infect. Microbiol..

[42] Cobos-Mayo M., Martín-Taboada A., Aliaga-Samanez A., Segura Moreno M., Olivero J. (2026). Global risk assessment of Lyme borreliosis transmission. Pathog. Glob. Health.

[43] Estrada-Peña A., Wijburg S.R., Sprong H. (2026). Climate driven patterns shape clusters of co-occurring ticks and vertebrates in the Western Palearctic-Tropics. Int. J. Parasitol..

[44] Bouchard C., Dumas A., Baron G., Bowser N., Leighton P.A., Lindsay L.R., Aenishaenslin C. (2023). Integrated human behavior and tick risk maps to prioritize Lyme disease interventions using a One Health approach. Ticks Tick Borne Dis..

[45] Estrada-Peña A., Cevidanes A., Sprong H., Millán J. (2021). Pitfalls in tick and tick-borne pathogens research, some recommendations and a call for data sharing. Pathogens.

[46] Lansdell S., Sharif M.S., Zorto A., Seto M., Negera E., Cutler S. (2025). Machine learning-based techniques for assessing critical factors for European tick abundance. Int. J. Comput. Theory Eng..

[47] Brunner J.L., LoGiudice K., Ostfeld R.S. (2008). Estimating reservoir competence of *Borrelia burgdorferi* hosts: Prevalence and infectivity, sensitivity, and specificity. J. Med. Entomol..

[48] van Wieren S.E., Hofmeester T.R. (2016). The role of large herbivores in *Ixodes ricinus* and *Borrelia burgdorferi* s.l. dynamics. Ecology and Prevention of Lyme Borreliosis.

[49] Zhao G.P., Wang Y.X., Fan Z.W., Ji Y., Liu M.J., Zhang W.H., Fang L.Q. (2021). Mapping ticks and tick-borne pathogens in China. Nat. Commun..

[50] Springer A., Kahl O., Król N., Pfeffer M., Chitimia-Dobler L., Lindau A., Strube C. (2026). Ticks in the landscape: Fragmentation impacts density of Europe’s principal tick-borne disease vector, *Ixodes ricinus*, across Germany. Ticks Tick Borne Dis..

[51] Spengler J.R., Estrada-Peña A. (2018). Host preferences support the prominent role of *Hyalomma* ticks in the ecology of Crimean-Congo hemorrhagic fever. PLoS Negl. Trop. Dis..

